# Long-term residual cardiovascular risk after acute coronary syndrome: antithrombotic treatment options

**DOI:** 10.1007/s12471-021-01604-4

**Published:** 2021-08-06

**Authors:** D. R. P. P. Chan Pin Yin, J. M. ten Berg

**Affiliations:** 1 Department of Cardiology, St. Antonius Hospital, Nieuwegein, The Netherlands; 2 Cardiovascular Research Centre, Maastricht, The Netherlands

**Keywords:** Acute coronary syndrome, Antithrombotic therapy, Ischaemic risk, Myocardial infarction

## Abstract

The residual risk of patients surviving until 1 year after acute coronary syndromes (ACS) is still high, despite secondary prevention. The cornerstone of treatment of patients with ACS is dual antiplatelet therapy (DAPT) consisting of low-dose aspirin and a P2Y12 inhibitor (clopidogrel, prasugrel or ticagrelor) for 12 months, or less in those patients at higher risk for bleeding. To reduce the residual risk beyond 1 year in those patients not at high bleeding risk who tolerated DAPT and did not suffer an (ischaemic or bleeding) event would intuitively mean to prolong DAPT. However, prolonged DAPT always comes at the cost of more bleeding. Therefore, assessing both ischaemic and bleeding risk in these patients at 1 year after ACS is crucial. In addition, another antithrombotic treatment consisting of low-dose rivaroxaban combined with low-dose aspirin has been shown to reduce ischaemic events. In this review, we describe residual thrombotic risk at 1 year after ACS, evaluate the evidence for antithrombotic options beyond 1 year and provide a practical guide to determine which patients would benefit the most from these therapies.

## Residual risk at 1 year after acute coronary syndrome

Over the past decades, the overall mortality of patients admitted with acute coronary syndrome (ACS) has decreased [1, 2]. Simultaneously, implementation of evidence-based treatments in ACS patients has led to a lower risk of recurrent ischaemic events [1, 2]. Also, with the use of newer generation drug-eluting stents (DES) [3], the incidence of both early and especially late [> 1 year after percutaneous coronary intervention (PCI)] stent thrombosis (ST), the most feared complication, has decreased. However, nationwide registries in different countries have also shown that a large proportion of patients have a recurrent cardiovascular event [i.e. myocardial infarction (MI), stroke or cardiovascular death] in the years following their initial admission [4, 5]. For example, a registry of ACS patients from the United Kingdom and Belgium has shown that one in five ACS patients have died after 5 years, 13% of them due to cardiovascular causes, implying that additional secondary prevention measures are warranted beyond the 1st year [4]. In addition, not only in registries, but even in randomised controlled trials (RCTs) where medical treatment is optimally controlled, the incidence of recurrent cardiovascular events is still high [6]. Extended antithrombotic strategies have been developed to reduce these long-term cardiovascular events, but all come at the cost of more bleeding [6–9]. However, it is important to acknowledge the different grades of severity in both bleeding and ischaemic events as, for example, not all moderate or severe bleeding events have the same impact on mortality as MI [10]. Therefore, a careful assessment should be made to select those patients who would benefit from such an intensified strategy based on both bleeding and residual ischaemic risk post-ACS [11, 12]. In this review, we discuss the evidence behind the different strategies of extended antithrombotic treatment and provide a practical guide to determine which patients would benefit the most from these therapies. As a consequence, we do not discuss the optimal antithrombotic therapy in the 1st year post-ACS, neither are other pathways, such as inflammation, the topic of this review.

## Extended dual antiplatelet treatment beyond 1 year

Several trials have studied the effect of longer dual antiplatelet therapy (DAPT) duration (i.e. beyond 12 months) in patients undergoing PCI [7, 13–15]. The DAPT study was the first placebo-controlled trial that showed superiority of continued DAPT to 30 months compared to standard DAPT of 12 months in terms of MI and ST rates (4.3% vs 5.9%, *p* < 0.001) [7]. However, an important increase in moderate or severe bleeding was seen in continued DAPT (2.5% vs 1.6%, *p* < 0.001), and concerns arose from the higher rates of non-cardiovascular death (1.0% vs 0.5%, *p* = 0.002). This increase in non-cardiovascular death was also shown in a meta-analysis of RCTs that compared short (3–12 months) to long (12–36 months) DAPT in patients treated with DES [hazard ratio (HR) 0.67, 95% confidence interval (CI) 0.51–0.89] [16]. In the trials included in this meta-analysis, over half of the patients had chronic coronary syndromes (CCS). Bare metal stents or first-generation DES were still frequently used and patients were predominantly treated with clopidogrel (and not the stronger agents ticagrelor or prasugrel) in addition to aspirin [16]. Thus, these trials may not adequately portray the contemporary treatment of patients admitted with ACS.

A better representation of the modern-day post-ACS patient is provided by the Prevention of Cardiovascular Events in Patients with Prior Heart Attack Using Ticagrelor Compared to Placebo on a Background of Aspirin–Thrombolysis in Myocardial Infarction 54 (PEGASUS-TIMI 54) trial, the largest RCT to date investigating extended DAPT use, although in this trial only a minority continued DAPT while most patients restarted DAPT [6]. In this trial, 21,162 patients who had had a MI 1–3 years earlier and had at least one additional high-risk feature (i.e. age ≥ 65 years, diabetes mellitus requiring medication, more than one prior MI, multivessel disease or renal impairment) were randomly assigned, in a double-blind 1:1:1 fashion, to ticagrelor 90 mg twice daily, 60 mg twice daily or placebo. All patients were on low-dose aspirin and were followed for a median of 33 months. Both ticagrelor doses reduced the composite of cardiovascular death, MI or stroke (at 3 years, 7.85% with ticagrelor 90 mg and 7.77% with ticagrelor 60 mg) as compared to 9.04% with placebo (HR for 90 mg 0.85, 95% CI 0.75–0.96, *p* = 0.008; HR for 60 mg 0.84, 95% CI 0.74–0.95, *p* = 0.004). Thrombolysis in myocardial infarction (TIMI) major bleeding was higher with ticagrelor (2.60% with 90 mg and 2.30% with 60 mg) than with placebo (1.06%) (*p* < 0.001 for each dose vs placebo). The rate of death from any cause was not reduced with ticagrelor. Intracranial haemorrhage or fatal bleeding in the three groups was 0.63%, 0.71% and 0.60%, respectively. Thus, because the decrease in thrombotic events and the increase in bleeding events were quite similar in magnitude, not all patients at 1 year post-MI should be treated with a longer duration of DAPT. An interesting subanalysis of the PEGASUS-TIMI 54 adds to the selection of patients that may benefit from extended-duration DAPT, demonstrating that patients who continue DAPT without interruption show a greater benefit than patients who restarted DAPT after an initial discontinuation 1 year post-MI [17].

A meta-analysis investigated 33,435 patients that had had a prior MI and were randomised to extended DAPT (low-dose aspirin plus clopidogrel, prasugrel or ticagrelor beyond 1 year) (*n* = 20,203) or to aspirin alone (*n* = 13,232) [14]. Extended DAPT decreased the composite of cardiovascular death, non-fatal MI and non-fatal stroke compared with aspirin alone [6.4% vs 7.5%; risk ratio (RR) 0.78, 95% CI 0.67–0.90; *p* = 0.001] and reduced cardiovascular death (2.3% vs 2.6%; RR 0.85, 95% CI 0.74–0.98; *p* = 0.03), with no increase in non-cardiovascular death (RR 1.03, 95% CI 0.86–1.23; *p* = 0.76). Extended DAPT also reduced MI (RR 0.70, 95% CI 0.55–0.88; *p* = 0.003), stroke (RR 0.81, 95% CI 0.68–0.97; *p* = 0.02), and ST (RR 0.50, 95% CI 0.28–0.89; *p* = 0.02). There was an increase in major bleeding (1.85% vs 1.09%; RR 1.73, 95% CI 1.19–2.50; *p* = 0.004) but not fatal bleeding (0.14% vs 0.17%; RR 0.91, 95% CI 0.53–1.58; *p* = 0.75). A second, aggregate meta-analysis on extended DAPT, which is more contemporary because it included only patients after DES implantation (*n* = 21,475), compared short (6–12 months) to extended (18–48 months) DAPT and stratified patients according to clinical presentation (CCS and ACS) [15]. In this analysis, similar results were found with increased rates of MI, ST and the composite of death, MI and stroke in patients with short DAPT, but higher rates of major bleeding and non-cardiac death in patients with extended DAPT [15]. However, this adverse effect was primarily driven by patients with CCS. Very important to the discussion about residual risk at 1 year post-ACS is that extended DAPT in ACS patients showed no significant increase in major bleeding (HR 0.94, 95% CI 0.48–1.81) and non-cardiac death (HR 0.94, 95% CI 0.59–1.52), and reduced the rates of death, MI, stroke and major bleeding combined (6.0% vs 4.4%, *p* < 0.0001) with a number needed to treat of 61 to achieve a net clinical benefit. These meta-analyses teach us that to be candidates for a longer duration of DAPT patients should at least have had a spontaneous MI or ACS, while the risk of bleeding due to extended DAPT seems to be less of an issue in ACS patients than in CCS patients. Consequently, the latest guidelines of the European Society of Cardiology (ESC) conclude that extended DAPT should be considered in ACS patients at high ischaemic risk without increased risk for major or life-threatening bleeding (class IIa, level of evidence A) [12].

## Long-term treatment with low-dose factor Xa inhibitor and aspirin

Oral anticoagulants reduce the risk of arterial thrombotic events. For example, in the Anti-Xa Therapy to Lower Cardiovascular Events in Addition to Standard Therapy in Subjects with Acute Coronary Syndrome–Thrombolysis in Myocardial Infarction 51 (ATLAS ACS 2‑TIMI 51) trial, additional low-dose rivaroxaban (2.5 mg twice daily) reduced the incidence of cardiovascular death, MI and stroke at 2 years post-ACS in patients mostly treated with low-dose aspirin and clopidogrel with a median duration of rivaroxaban use of 13.3 months. However, an important increase in bleeding, but not fatal bleeding, was found [8]. In a meta-analysis of patients with recent ACS and treated with DAPT, the addition of direct oral anticoagulant (DOAC) drugs to antiplatelet therapy led to a modest reduction of cardiovascular events (HR 0.87, 95% CI 0.80–0.95), but more than doubled the risk of major bleeding (HR 2.34, 95% CI 2.06–2.66) [18]. The ischaemia/bleeding risk trade-off was better in patients with additional DOAC use and aspirin alone. In the years following these trials, the Cardiovascular Outcomes for People Using Anticoagulation Strategies (COMPASS) trial was completed, which may be more relevant to the discussion about how to treat residual risk at 1 year. In this double-blind trial, 27,395 patients with stable coronary syndromes or peripheral artery disease received rivaroxaban (2.5 mg twice daily) plus low-dose aspirin, rivaroxaban (5 mg twice daily) or low-dose aspirin [9]. The composite of cardiovascular death, stroke or MI occurred less often with rivaroxaban plus aspirin than with aspirin alone (4.1% vs 5.4%; HR 0.76, 95% CI 0.66–0.86; *p* < 0.001), with the greatest effect in the reduction of stroke (HR 0.58, 95% CI 0.44–0.76). Concurrently, major bleeding occurred more with rivaroxaban plus aspirin (3.1%) than with aspirin alone (1.9%; HR 1.70, 95% CI 1.40–2.05; *p* < 0.001). There was no significant difference in intracranial or fatal bleeding with rivaroxaban plus aspirin. Death occurred in 3.4% with rivaroxaban plus aspirin as compared with 4.1% with aspirin alone (HR 0.82, 95% CI 0.71–0.96; *p* = 0.01; threshold *p*-value for significance 0.0025). The rate of the primary outcome was not significantly lower with rivaroxaban alone than with aspirin alone, but major bleeding occurred more with rivaroxaban alone. The study was stopped because of the superiority of rivaroxaban plus aspirin after a mean follow-up of 23 months. Greater absolute risk reductions with rivaroxaban plus aspirin were found in patients at high ischaemic risk (e.g. both coronary artery disease and peripheral artery disease or concomitant diabetes) [19]. Although patients with CCS and not ACS were included in the COMPASS trial, a large group of patients (62%) had a prior MI. Therefore, the 2020 ESC guidelines for the management of ACS patients without persistent ST-segment elevation state that rivaroxaban 2.5 mg twice daily in addition to aspirin should be considered in patients at high thrombotic risk and without an increased risk for major or life-threatening bleeding, and may be considered in patients with a moderately high thrombotic risk [12].

## How to select patients benefitting from extended antithrombotic treatment beyond 1 year post-ACS

**Fig. 1:**
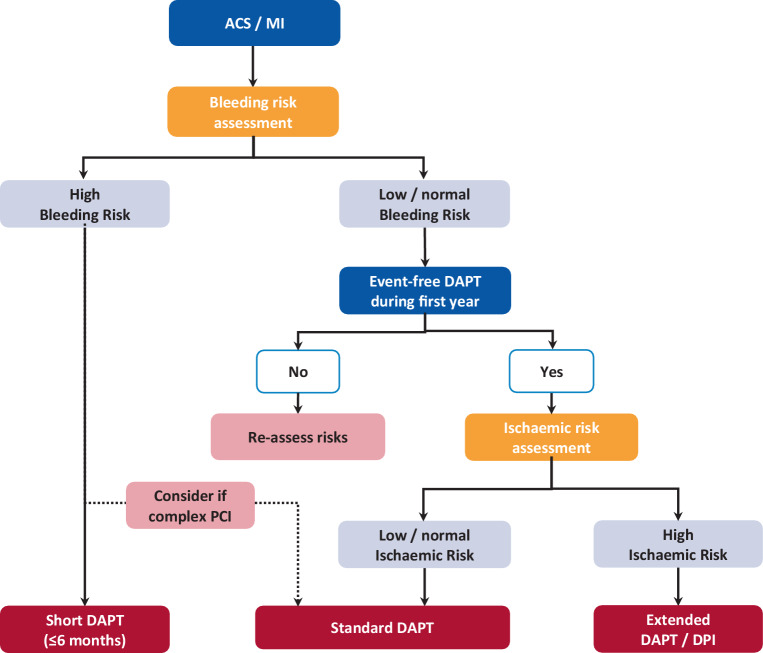
Flowchart of bleeding and ischaemic risk assessment following acute coronary syndrome (*ACS*) or myocardial infarction (*MI*). Bleeding risk should be assessed during hospital admission. Bleeding risk is assessed by use of the PRECISE-DAPT score or the ARC-HBR criteria [20, 21]. In patients at high bleeding risk, a short duration (≤ 6 months) of dual antiplatelet therapy (*DAPT*) should be considered. In patients at high bleeding risk and with a complex percutaneous coronary intervention (*PCI*), the standard DAPT duration may be considered. In patients without a high bleeding risk and who have tolerated DAPT in the first 12 months, the ischaemic risk should be assessed (see Tab. 2). In patients at high ischaemic risk extended DAPT or dual pathway inhibition (*DPI*) should be considered

Although the above literature clearly shows that there is evidence for extended antithrombotic therapy at 1 year post-ACS in patients with a higher thrombotic risk, we still struggle with how to define this ‘higher thrombotic risk’, how to balance the increased bleeding risk (many patients with a higher thrombotic risk also have a higher bleeding risk) and finally how to choose between continuing DAPT or switching to low-dose DOAC added to aspirin. Nevertheless, below we give our personal preference and describe how we treat residual antithrombotic risk in daily practice. An overview of the options for extended dual antithrombotic therapy is listed in Tab. 1. It should be noted that the numbers needed to treat or to harm listed for either ischaemic or bleeding events are derived from the original studies without initial risk stratification. In practice, careful risk assessment should be made first before extending dual antithrombotic therapy in patients with ACS [11, 12]. This risk stratification should involve both thrombotic and bleeding risk, taking into account clinical, anatomical and procedural characteristics. Risk stratification should start at discharge post-ACS and risk scores favoured by the ESC guidelines are the PREdicting bleeding Complications In patients undergoing Stent implantation and subsEquent Dual AntiPlatelet Therapy (PRECISE-DAPT) and the Academic Research Consortium High Bleeding Risk (ARC-HBR) score (Fig. 1; [12, 20, 21]). Our personal preference is the PRECISE-DAPT score, as this is better validated than other scores [22]. Further, the ARC-HBR score was not developed to tailor DAPT duration. The PRECISE-DAPT score (consisting of the variables haemoglobin, age, creatinine clearance, white blood cell count and previous spontaneous bleeding) can be used to reduce the duration of DAPT to less than the standard 1 year [20]. In patients with a high PRECISE-DAPT score (≥ 25), standard 1‑year DAPT is associated with no reduction in ischaemic events, but with a strong increase in bleeding [20]. These high bleeding risk patients should receive shorter DAPT (≤ 6 months), even those patients with a concomitant high ischaemic risk [23]. However, in selected patients with complex PCI (e.g. bifurcation stenting, ST), 12 months of DAPT may be considered after consultation with the interventional cardiologist who performed the PCI (Fig. 1). Patients without a high bleeding risk (e.g. PRECISE-DAPT score < 25) should be treated with standard 1‑year DAPT. Further, residual thrombotic risk should be assessed at 1 year in all patients that tolerated DAPT and did not suffer a thrombotic or bleeding event (Fig. 1). The current guidelines advise the use of either the DAPT score, the criteria used in the PEGASUS-TIMI 54 study or the criteria for high ischaemic risk in the ESC guidelines, which consist of clinical and angiographical/procedural risk factors (i.e. diabetes mellitus requiring medication, polyvascular disease, bifurcation stenting etc.), to select patients that may benefit from extended DAPT beyond 1 year post-ACS (Tab. 2; [11, 12]). The DAPT score is a combined score of both ischaemic and bleeding risk (Tab. 2; [24]). In patients with a high score (≥ 2), extended DAPT (12–30 months) resulted in reductions in the incidence of MI or ST [absolute risk difference (ARD) −3.0%; 95% CI −4.1 to −2.1, *p* < 0.001] with a number needed to treat of 34, and no increase in moderate or severe bleeding (ARD +0.4%; 95% CI −0.3% to +1.0%, *p* = 0.26) with a number needed to harm of 272. In patients with a low score (< 2), extended DAPT was associated with an increase in moderate or severe bleeding events, without reductions in ischaemic events. Thus, only patients with a high DAPT score should be treated with extended DAPT. Also, the PEGASUS-TIMI 54 inclusion criteria could be used (Tab. 2). A third option is to apply the criteria for high ischaemic risk in the latest ESC guidelines (Tab. 2; [12]). These criteria were selected based on the combined evidence of the COMPASS, DAPT and PEGASUS TIMI 54 studies in addition to observational evidence from large observational registries [6, 7, 9, 25–27]. Our personal preference is the DAPT score because it is easy to use and can be calculated at discharge. Thus it does not take up extra time at a busy outpatient clinic when the decision regarding the continuation of DAPT has to be made together with the patient. It is noteworthy that the ESC criteria have not yet been validated externally, as opposed to the DAPT score [28] and the PEGASUS-TIMI 54 criteria [29].

**Table 1:** Antithrombotic treatment options for extended therapy beyond 1 year after an acute coronary syndrome (*ACS*). Aspirin dose is 75–100 mg once per day in all strategies. All strategies should be applied only if the bleeding risk is low (e.g. PRECISE-DAPT score < 25, ARC-HBR criteria not met)

Extended dual antithrombotic treatment	Dose of additional drug	Continue/start treatment	Eligible patients	NNT (ischaemic events)	NNH (bleeding events)
Aspirin + clopidogrel	75 mg, once per day	At 1 year post-ACS	One year uneventful DAPT use	63	105
Aspirin + prasugrel	5^a^/10 mg, once per day	At 1 year post-ACS + PCI	One year uneventful DAPT use	63	105
Aspirin + ticagrelor	60/90 mg, twice per day	At 1 year post-ACS	One year uneventful DAPT use	84	81
Aspirin + rivaroxaban	2.5 mg, twice per day	At 1 year or more post-ACS	High residual ischaemic risk^b^	77	84

^a^The reduced prasugrel dose is only for patients with a body weight below 60 kg or age above 75 years

^b^For criteria for high ischaemic risk, see Tab. 2

*ARC-HBR* Academic Research Consortium for High Bleeding Risk, *DAPT* dual antiplatelet therapy, *NNH* number needed to harm, *NNT* number needed to treat, *PCI* percutaneous coronary intervention, *PRECISE-DAPT* PREdicting bleeding Complications In patients undergoing Stent implantation and subsEquent Dual AntiPlatelet Therapy

**Table 2:** Risk scores and criteria for high ischaemic risk and eligibility for extended dual antithrombotic therapy. Use these criteria at 1 year after acute coronary syndrome (*ACS*) or myocardial infarction (*MI*). MI is defined as spontaneous MI. Multivessel coronary artery disease (*CAD*) is defined as stenosis of ≥ 50% in two major coronary territories (i.e. left anterior descending artery, intermediate artery, left circumflex artery, right coronary artery, left main coronary artery, a major branch or bypass graft), including revascularised arteries

Risk score/criteria	DAPT score		PEGASUS-TIMI 54 criteria	High ischaemic risk—ESC 2020
Variables	Age: ≥ 75 years	−2 pts	*Prior MI last 1–3 years* and *Age: ≥* *50 years* and *≥* *1 of the following*: – Age: ≥ 65 years – DM requiring medication – A second prior MI – Multivessel CAD – CKD with eGFR < 60 ml/min per 1.73 m^2^	*Complex CAD*^a^ and *≥* *1 of the following*: – Risk enhancers: DM requiring medication History of recurrent MI Any multivessel CAD Polyvascular disease (CAD + PAD) Premature (< 45 years)/accelerated^b^ CAD Systemic inflammatory disease^c^ CKD with eGFR 15–59 ml/min – Technical aspects: At least 3 stents implanted At least 3 lesions treated Total stent length < 60 mm History of complex revascularisation^d^ History of ST on antiplatelet treatment
	Age: 65–74 years	−1 pt		
	Age: ≤ 64 years	0 pt		
	Smoking (last 2 years)	+1 pt		
	Diabetes mellitus	+1 pt		
	MI at presentation	+1 pt		
	Prior PCI or prior MI	+1 pt		
	Stent diameter < 3 mm	+1 pt		
	CHF or LVEF < 30%	+2 pts		
	Vein graft stenting	+2 pts		
High ischaemic risk defined as:	*Total DAPT score: ≥* *2*		*All 3 criteria should be met*	*Complex CAD +1 or more criteria (risk enhancer or technical aspect) should be met*

^a^Complex CAD is based on individual clinical judgement with knowledge of patients’ cardiovascular history and/or coronary anatomy

^b^Accelerated CAD is defined as a new lesion within a 2-year timeframe

^c^For example, human immunodeficiency virus, systemic lupus erythematosus, chronic arthritis

^d^Left main stenting, bifurcation stenting with ≥ 2 stents implanted, chronic total occlusion, stenting of last patent vessel

*CHF* congestive heart failure, *CKD* chronic kidney disease, *DAPT* dual antiplatelet therapy, *DM* diabetes mellitus, *eGFR* estimated glomerular filtration rate, *ESC* European Society of Cardiology, *LVEF* left ventricular ejection fraction, *PAD* peripheral artery disease, *PEGASUS-TIMI 54* Prevention of Cardiovascular Events in Patients with Prior Heart Attack Using Ticagrelor Compared to Placebo on a Background of Aspirin–Thrombolysis in Myocardial Infarction 54, *PCI* percutaneous coronary intervention, *ST* stent thrombosis

Finally, to assess whether patients with a high thrombotic risk benefit more from extending DAPT or combining low-dose rivaroxaban with aspirin is not possible, as these therapies have not been compared head-to-head. Intuitively, one would keep the patient on DAPT when this is well tolerated and start low-dose rivaroxaban in a patient with a high thrombotic risk treated with aspirin alone. We start low-dose rivaroxaban in patients with characteristics that are in line with the inclusion criteria of the COMPASS trial, especially those with peripheral arterial disease and/or carotid artery disease. Both long-term DAPT with low-dose ticagrelor (60 mg b.i. d.) and low-dose rivaroxaban (2.5 mg b.i. d.) with aspirin have proven to be cost-effective compared to aspirin alone [30, 31]. Discontinuation rates were higher for low-dose ticagrelor in the PEGASUS-TIMI-54 trial compared to the COMPASS trial (29% at 33 months and 16.5% at a mean follow-up duration of 23 months, respectively) [9, 32]. Low-dose ticagrelor was discontinued due to adverse events in 16% of patients, mainly because of non-major bleeding (6.2%) and dyspnoea (4.6%); in the majority this occurred early after randomisation. Details on rates and reasons for discontinuation in the COMPASS trial have not been published. The duration of extended treatment should depend on the patients’ tolerance. The current literature only supports the use of extended dual antithrombotic therapy from 2 to 4 years post-ACS or post-MI, but some patients at a continuous high ischaemic risk may have an indication for lifelong intensified antithrombotic treatment. It is advised that information on any bleeding complications while on dual antithrombotic therapy should be obtained in the outpatient setting and that patients’ bleeding risk and ischaemic risk should be reassessed yearly.

## References

[1] Szummer K, Wallentin L, Lindhagen L (2017). Improved outcomes in patients with ST-elevation myocardial infarction during the last 20 years are related to implementation of evidence-based treatments: experiences from the SWEDEHEART registry 1995–2014. Eur Heart J.

[2] Szummer K, Wallentin L, Lindhagen L (2014). Relations between implementation of new treatments and improved outcomes in patients with non-ST-elevation myocardial infarction during the last 20 years: experiences from SWEDEHEART registry 1995 to. Eur Heart J.

[3] Byrne RA, Joner M, Kastrati A (2014). Stent thrombosis and restenosis: what have we learned and where are we going? The Andreas Grüntzig Lecture ESC. Eur Heart J.

[4] Fox KAA, Carruthers KF, Dunbar DR (2010). Underestimated and under-recognized: the late consequences of acute coronary syndrome (GRACE UK-Belgian Study). Eur Heart J.

[5] Jernberg T, Hasvold P, Henriksson M, Hjelm H, Thuresson M, Janzon M (2015). Cardiovascular risk in post-myocardial infarction patients: nationwide real world data demonstrate the importance of a long-term perspective. Eur Heart J.

[6] Bonaca MP, Bhatt DL, Cohen M (2015). Long-term use of ticagrelor in patients with prior myocardial infarction. N Engl J Med..

[7] Mauri L, Kereiakes DJ, Yeh RW (2014). Twelve or 30 months of dual antiplatelet therapy after drug-eluting stents. N Engl J Med..

[8] Mega JL, Braunwald E, Wiviott SD (2012). Rivaroxaban in patients with a recent acute coronary syndrome. N Engl J Med..

[9] Eikelboom JW, Connolly SJ, Bosch J (2017). Rivaroxaban with or without aspirin in stable cardiovascular disease. N Engl J Med..

[10] Valgimigli M, Costa F, Lokhnygina Y (2017). Trade-off of myocardial infarction vs. bleeding types on mortality after acute coronary syndrome: lessons from the Thrombin Receptor Antagonist for Clinical Event Reduction in Acute Coronary Syndrome (TRACER) randomized trial. Eur Heart J.

[11] Valgimigli M, Bueno H, Byrne RA (2017). ESC focused update on dual antiplatelet therapy in coronary artery disease developed in collaboration with EACTS: the Task Force for dual antiplatelet therapy in coronary artery disease of the European Society of Cardiology (ESC) and of the European. Eur Heart J.

[12] Collet J‑P, Thiele H, Barbato E (2021). ESC Guidelines for the management of acute coronary syndromes in patients presenting without persistent ST-segment elevation. Eur Heart J..

[13] Valgimigli M, Campo G, Monti M (2012). Short-versus long-term duration of dual-antiplatelet therapy after coronary stenting: a randomized multicenter trial. Circulation..

[14] Udell JA, Bonaca MP, Collet JP (2016). Long-term dual antiplatelet therapy for secondary prevention of cardiovascular events in the subgroup of patients with previous myocardial infarction: A collaborative meta-analysis of randomized trials. Eur Heart J.

[15] Palmerini T, Bruno AG, Gilard M (2019). Risk-benefit profile of longer-than-1-year dual-antiplatelet therapy duration after drug-eluting stent implantation in relation to clinical presentation. Circ Cardiovasc Interv.

[16] Palmerini T, Benedetto U, Bacchi-Reggiani L (2015). Mortality in patients treated with extended duration dual antiplatelet therapy after drug-eluting stent implantation: a pairwise and Bayesian network meta-analysis of randomised trials. Lancet.

[17] Bonaca MP, Bhatt DL, Steg PG (2016). Ischaemic risk and efficacy of ticagrelor in relation to time from P2Y12 inhibitor withdrawal in patients with prior myocardial infarction: insights from PEGASUS-TIMI 54. Eur Heart J.

[18] Oldgren J, Wallentin L, Alexander JH (2013). New oral anticoagulants in addition to single or dual antiplatelet therapy after an acute coronary syndrome: a systematic review and meta-analysis. Eur Heart J.

[19] Anand SS, Eikelboom JW, Dyal L (2019). Rivaroxaban plus aspirin versus aspirin in relation to vascular risk in the COMPASS trial. J Am Coll Cardiol..

[20] Costa F, van Klaveren D, James S (2017). Derivation and validation of the predicting bleeding complications in patients undergoing stent implantation and subsequent dual antiplatelet therapy (PRECISE-DAPT) score: a pooled analysis of individual-patient datasets from clinical trials. Lancet.

[21] Urban P, Mehran R, Colleran R (2019). Defining high bleeding risk in patients undergoing percutaneous coronary intervention: a consensus document from the Academic Research Consortium for High Bleeding Risk. Eur Heart J.

[22] Pin Yin CD, Azzahhafi J, James S (2020). Risk assessment using risk scores in patients with acute coronary syndrome. J Clin Med.

[23] Costa F, Van Klaveren D, Feres F (2019). Dual antiplatelet therapy duration based on ischemic and bleeding risks after coronary stenting. J Am Coll Cardiol..

[24] Yeh RW, Secemsky EA, Kereiakes DJ (2016). Development and validation of a prediction rule for benefit and harm of Dual antiplatelet therapy beyond 1 year after percutaneous coronary intervention. JAMA.

[25] Bhatt DL, Eagle KA, Ohman EM (2010). Comparative determinants of 4‑year cardiovascular event rates in stable outpatients at risk of or with atherothrombosis. JAMA.

[26] Darmon A, Sorbets E, Ducrocq G (2019). Association of multiple enrichment criteria with ischemic and bleeding risks among COMPASS-eligible patients. J Am Coll Cardiol..

[27] Collet J‑P, Zeitouni M, Procopi N (2019). Long-term evolution of premature coronary artery disease. J Am Coll Cardiol..

[28] Witberg G, Zusman O, Yahav D, Perl L, Vaknin-Assa H, Kornowski R (2019). Meta-analysis of studies examining the external validity of the DAPT score. Eur Heart J Cardiovasc Pharmacother.

[29] Cosentino N, Campodonico J, Faggiano P (2019). A new score based on the PEGASUS-TIMI 54 criteria for risk stratification of patients with acute myocardial infarction. Int J Cardiol..

[30] Magnuson EA, Li H, Wang K (2017). Cost-effectiveness of long-term ticagrelor in patients with prior myocardial infarction: results from the PEGASUS-TIMI 54 trial. J Am Coll Cardiol..

[31] Zomer E, Si S, Hird TR (2019). Cost-effectiveness of low-dose rivaroxaban and aspirin versus aspirin alone in people with peripheral or carotid artery disease: an Australian healthcare perspective. Eur J Prev Cardiol.

[32] Bonaca MP, Bhatt DL, Oude Ophuis T (2016). Long-term tolerability of ticagrelor for the secondary prevention of major adverse cardiovascular events: a secondary analysis of the PEGASUS-TIMI 54 trial. JAMA Cardiol.

